# Human Skin Pigmentation: From a Biological Feature to a Social Determinant

**DOI:** 10.3390/healthcare11142091

**Published:** 2023-07-22

**Authors:** Sarah Mosca, Aldo Morrone

**Affiliations:** 1 Laboratory of Cutaneous Physiopathology and Integrated Center of Metabolomics Research, San Gallicano Dermatological Institute, IRCCS, 00144 Rome, Italy; 2 Scientific Direction, San Gallicano Dermatological Institute, IRCCS, 00144 Rome, Italy

**Keywords:** ethnicity, skin colour, pigmentary disorders, pigmentation, health inequalities

## Abstract

Skin pigmentation is the most variable human characteristic that can be observed and has been used throughout history to classify humans into distinct groups. Many factors influence skin colour, but the melanin pigment is considered the most important because its type and quantity can determine variations in pigmentation shades. The evolution of skin pigmentation started around 1.6–2 million years ago. As a result of migratory phenomena to places with less ultraviolet radiation (UVR) and other seasonal regimes, the selection of depigmented skin and different tanning capabilities occurred over time. Thus, genetic adaptation to new environmental conditions gradually led to changes in skin pigmentation. Despite the biological importance of pigmentation, variation in skin colour has led to social and health inequalities. Since Linnaeus, skin colour classifications have been used to describe different human groups, encouraging the misuse of a biological characteristic. This review examines the characterisation of pigmentation and its evolution through history and society. The unequal perception of pigmentation diversity has led to an incomplete state of dermatological training and issues in medical approach in dermatology. The consciousness of all these aspects increases the need to address and overcome dermatologic and social health disparities related to skin pigmentation.

## 1. Introduction

Skin pigmentation is the first variable and observable feature of human beings. The perception of the pigmentation variation has changed over time and has often been used to construct a “racial classification”. The 17–18th centuries represented the beginning of the distinction of people according to “races”, with the concept of the primacy of whiteness, legitimising a permanent and hereditary condition of slavery for races considered inferior. People with highly pigmented skin were called “Negroes”, as a separate race, thus implying that Europeans, considered fair-skinned individuals, were the master race [1].

Some scientists and anthropologists were influenced by racism over time and tried to justify it biologically by several techniques such as the craniometric analysis, the measure of the human skull, that had to prove the biological superiority of fair-skinned people over individuals with highly pigmented skin [2]. An example case to understand the nature of scientific racism is represented by the naturalist Louis Agassiz that, in the 19th century, tried to support the “polygenism” theory, which argued that human races were distinct species. Agassiz explained the differences between “Whites” and “Blacks” as the result of fixed intrinsic distinction due to the separate creations of the races [3]. Thus, racism had many faces. In the 19–20th century, a particular example was represented by Italian immigrants, mainly darker-skinned people from southern Italy, in the United States of America. Italians were described as “swarthy” or “kinky-haired”, and called epithets like “dago” and “guinea”, terms of derision applied to enslaved Africans and their descendants [4].

The vision of races became the basis of the propaganda and the eugenic studies around the 19th–20th century with the climax in the Nazi Aryan ideal [2,5]. After the tragedy and the horror of the Nazis, scientists tried to change the distorted concept of races, but racism has been difficult to eradicate. A recent example is the anthropologist Carleton Stevens Coon, who published “The Origin of Races” in 1962, a work where he supported the idea that some races had evolved from *Homo erectus* to *Homo sapiens* earlier than others [5]. This represented a resurgence of scientific racism and supported “white supremacy” again. 

Today, the scientific community seems to have learned from its own failures and is trying to completely overcome human prejudices and all forms of racism, including the scientific one, which is always a debated argument. One of the most important merits of the human genome project (The 1000 Genomes Project Consortium, 2000) has been to demonstrate the incredible gene similarity of all human beings, sweeping away any inferences about racial diversity. Still, today, scientific racism is a heated debate in the scientific community, and many recent studies have investigated the evolution of the relationship between pigmentation and dermatology. 

The first aim of this review is to explore what we know today about skin pigmentation, its history and how it is considered in medicine. The second one is also to trace the recent investigations describing a potential relationship between skin pigmentation and some cutaneous disorders. We conclude with a discussion about the actual situation of health disparities in dermatology.

## 2. Materials and Methods

We performed a systemic search of Pubmed and MeSH databases using the following terms: color skin, white skin, black skin, skin pigmentation, ethnicity, ethnic diseases, skin cancer, hyperpigmentation disorder, hypopigmentation disorder, skin disorders in combination with melanin, health disparities, evolution, scientific racism, race, inequalities, social determinant. Other articles were identified by checking reference lists of all articles that satisfied the criteria. Searches were conducted for all English peer-reviewed articles, with a preference for more recent literature.

## 3. The Role of Melanin in Skin Pigmentation

Constitutive skin pigmentation is mainly related to the amount and type of melanin, a pigment determined genetically for type and level [6]. Melanin, from the Greek word for black (μελαζ), has a key role in pigmentation and is produced by highly specialized cutaneous cells called melanocytes. These cells, distributed at the epidermal–dermal junction, are surrounded by keratinocytes (with a ratio of 1 melanocyte:36 keratinocytes) and look like dendritic cells containing organelles called melanosomes, crucial for the synthesis, storage, and transport of melanin outside [7].

Data in the literature suggest that the number of melanocytes is quite constant in all pigmented skins, whereas the size and distribution of melanosomes seem to have a role in the final appearance of human skin colour. Highly pigmented skin was found to contain up to five times more melanosomes, which are also bigger and more dispersed in the overall epidermis than those contained in fair skin [8]. In addition, in highly pigmented skin, melanosomes tend to be transferred individually to the surrounding keratinocytes, whereas melanosomes of fair skin are transferred in membrane-bound clusters (Figure 1) [9].

Melanin polymer is synthesized from oxidative tyrosine derivates and has two possible forms the ratio of which determines the pigmentation: the black-to-brown eumelanin and the less effective filter yellow-to-reddish pheomelanin that is found at high levels mainly in people with pale skin and red or fair hair [10]. 

Constitutive pigmentation can be modified by the tanning response induced by sun exposure (facultative pigmentation) as human adaption to different levels of seasonal UV [11]. Indeed, the melanogenesis process is activated by UVR, with melanin as the most important photoprotective agent against the UV-deleterious effects, such as DNA damage and oxidative stress [12]. 

Furthermore, melanin also has a crucial role in the defence against UVR-dependent photolysis of folic acid. Folic acid is essential for several cellular processes but is known mainly for its crucial role in pregnancy because its lack leads to a high risk of neural tube alterations [13]. Thus, a high melanin amount is able to absorb UVR which would otherwise penetrate deeply into the dermis, interfering with the normal synthesis of folic acid [14].

In light of all these aspects, skin pigmentation results a complex process that is influenced by several factors, such as genetics, melanosome morphology and melanin type, but can also be modified by events such as UV exposure. Disruption of these balances can consequently lead to pigmentary disorders.

## 4. The Evolution of Skin Pigmentation through Nudity

Skin pigmentation represents a variable characteristic that has evolved with human migrations and the need to adapt to new environmental and cultural conditions. There are several theories about human evolution, but most consider that hominins originated from African ancestors in the late Pliocene about 7–8 million years ago that had fair skin below thick black hairs, similar to chimpanzees that live today [15].

About 3–4 million years ago, because of an increase in dryness in Africa after the Earth’s climate change, hominins were forced to abandon their life on trees to move to open habitats with greater exposure to sun rays and more activity with movement which led to a risk of overheating [15,16]. Fossil foot bones from *Australopithecines* confirm that the upright position dates back to 4 million years ago when they presumably began to run on two legs for long distances up to 10 km [17]. As suggested by the physiological models of Wheeler [18] and Christophers [19], the loss of hair might be a consequence of bipedality. These models show that the increase in the distances covered by running and walking increased heat loss and required the improvement of sweat capacity. Consequentially, 1.6–2 million years ago, *Homo ergaster* evolved with a loss of body hair and the generation of eccrine sweat glands [16,18].

The direct exposure of skin to sun rays and the need to improve thermoregulation also led humans to gradually evolve the skin pigment [16]. The movement of *Homo erectus* out of Africa around 1.9 million years ago is the first important, but yet debated theorized event in human migrations. Recent data in the literature indicate that *Homo erectus* preferentially occupied areas with low or middle latitudes and with warm climates [20]. Then, *Homo sapiens* presumably represent the first population that moved to non-tropical latitudes out of Africa and Eurasia. The human fossils found outside Africa are dated to around 120,000–90,000 years ago at Levantine sites of Skhul and Qafzeh (Israel), but a recent fossil of human jawbone discovered at Misliya Cave, still in Israel, is traced back to around 170,000–194,000 years ago, suggesting that *Homo sapiens* moved out of Africa earlier than expected [21]. When hominins occupied new territories, the pigmentation gradually evolved and changed to adapt to different UV ray intensities and to protect from heat dispersion [15]. The evolution of human skin pigmentation included the association with genetic variants, as for genes *SLC24A5*, *SLC45A2*, *MC1R*, *TYR*, *TYRP1*, and *OCA2*, which led to a positive selection in European and Asian populations [22]. On the other hand, outside the tropics, with low and seasonal exposures to sunlight, highly pigmented skin prevented the UV-dependent generation of vitamin D_3_, an intraepidermal process essential for calcium metabolism. For this reason, genetic evolution was necessary to adapt to new environmental conditions. 

In conclusion, skin pigmentation is the result of several needs of human beings that evolved with them: it is not only a “shield” against UVR damages, but also an important defence that avoids heat dissipation and vitamin D deficiency.

## 5. Classifications of Human Beings through Colours of Skin

The need to classify patients has always been constant in dermatology to transform descriptions of medical diagnoses or procedures into a standardized statistical approach.

In the 18th century, when Europeans were considered superior to all other populations, the naturalist Carl Nilsson Linnaeus (1707–1778), with his work *Systema Naturae* (1735), was the first scientist who classified human beings according to most visible characteristics such as skin colour. He provided a systemic organization of the species, differentiating people into four groups: *Homo Europaeus albescens* (white and ingenious)*, Homo Americanus rubescens* (red and choleric)*, Homo Asiaticus fuscus* (yellow and melancholy), and *Homo Africanus niger* (black and phlegmatic). Linnaeus associated the feature of skin colour with human temper, and also described clothing and the system of government: Americans painted the body, Europeans wore tight-fitting clothes, Asians wore baggy robes, and Africans sprinkled themselves with grease.

Subsequently, the first to propose a classification of humanity based only on visible external characteristics was Johan Friedrich Blumenbach (1752–1840) in his *De generis humani varietate nativa* (1776). He used the term “Caucasian” as an anthropological definition for the first time, referring to the celebrated beauty of people who lived in the Mount Caucasus region. The term Caucasian was indeed born with Jean Chardin, a French traveller and jeweller who crossed the Caucasus Mountains in the 17th century, and in his book, *The Travels of Sir John Chardin*, described Western people of the Georgia region as “the most beautiful”, like angels [23].

Therefore, Blumenbach distinguished five varieties from different world areas: *Caucasian* (white skin), which he considered as individuals closest to the original ideal; *Mongolian* (yellow skin), *Ethiopian* (black skin), *American* (copper-coloured skin), and *Malay* (tawny-coloured skin) [24]. Even if Blumenbach never used the term “race”, he classified human beings in relation to the colour of their skin. 

Hence, after Linnaeus and Blumenbach, the observation that skin of any pigmentation tone has a specific response to environmental or physical stimulations led to several human skin classifications in medicine over time. 

Nowadays, the Fitzpatrick Skin Phototype Classification is the most accredited system. Developed in 1975, the Fitzpatrick scale classified skin phototypes as skin types on the basis of their ability to burn and tan after UV radiation. Initially, it considered four skin types, but it was modified later, in 1988, to also include darker skin tones [25]. Currently, it consists of six groups (Figure 2): 

I—Very Fair: always burns, never tans;

II—Fair: usually burns, tans with difficulty;

III—Medium: sometimes burns, usually tans;

IV—Olive: rarely burns, always tans;

V—Brown: rarely burns, easily tans;

VI—Black: never burns, always tans.

Even if this classification is easy to use and is accepted by dermatologists, its validity for all pigmentation tones is controversial. Several graduations of pigmentation are not fully considered and included in the phototypes scale. In addition, some physicians find this scale useful to describe “skin colours”, evidencing a misuse of this classification [26]. Therefore, even if the concept of “race” was created by society and not by biology, researchers and physicians constantly wonder about the ethics of using the Fitzpatrick scale, which is still the most widely used in dermatology.

To find alternatives to the Fitzpatrick scale, the scientific community is working on new approaches to overcoming its limitations. On this basis, Sharma et al. modified the Fitzpatrick scale for the Indian population by utilizing the reflectance spectrophotometry as a more objective tool for skin colour classification and erythema measure. This method was found to be accurate, but too expensive [27]. Recently, Dadzie et al. instead presented a novel scale, the “Eumelanin Human Skin Colour Scale” (EHSCS), a five-point classification related to published skin reflectance measurements. The authors sought to describe objectively the constitutive colour of human skin using eumelanin as the central descriptive and biological term, without social or racist connotations [28]. The EHSCS scale has limitations that the authors themselves point out, but it offers an opportunity to create other classifications that can consider skin properties besides colour. Other recent studies have evaluated the validity of self-reported skin colour using skin tone scales designed for specific populations. Japanese research established a self-reported skin colour assessment. Nakashima et al. observed a slight correlation between self-reported skin colour according to a selected skin colour chart and melanin index measured by spectrophotometry, but with recognized limitations for people with erythematous skin and the narrow range of skin colours considered [29].

All studies in the literature on new emerging skin classifications highlight the wide variety of shades of skin colour. This is also the message behind the internationally acclaimed the Humanae project created by the award-winning Brazilian photographer Angélica Dass. Her project began in 2014 and aimed to collect portraits of people with different skin colour shades observed worldwide. Dass added the skin colour identified on the background of each portrait after matching it with the *Pantone* industrial pallet via a computer algorithm [30]. Currently, more than 4000 images captured in more than 20 countries are in the project. 

The Humanae project demonstrates that dermatological skin colour classifications need to be enriched or developed with more inclusive and appropriate methods. Fortunately, the scientific community is working toward this aim. 

## 6. Skin Pathologies: The Role of Pigmentation

Data in the literature show a possible correlation between the prevalence and clinical manifestations of some skin diseases and skin pigmentation. 

### 6.1. Vitamin D Deficiency

Vitamin D production depends on the melanin amount in the skin because of the melanin’s crucial role in the absorption and the scattering of UVB and the consequent conversion of 7-DHC to pre-vitamin D3. Low vitamin D3 synthesis leads to vitamin D deficiency and data in the literature discuss the major prevalence of this disease in highly pigmented skin populations, such as African Americans [31]. These observations are presumably a consequence of historical events, such as the fast and constrained deportation of Africans to the Americas during the 16th–18th centuries and of more recent migratory flows, all towards places with higher latitudes and reduced sun exposure [32].

Fortunately, vitamin D deficiency could be treated with supplements or a targeted diet. Thus, Ames et al. debated about the presence of health disparities and life inequalities for African American people, with a consequent lesser awareness of the possibilities and health solutions they could have [31].

### 6.2. Postinflammatory Hyperpigmentation

**Figure 3:**
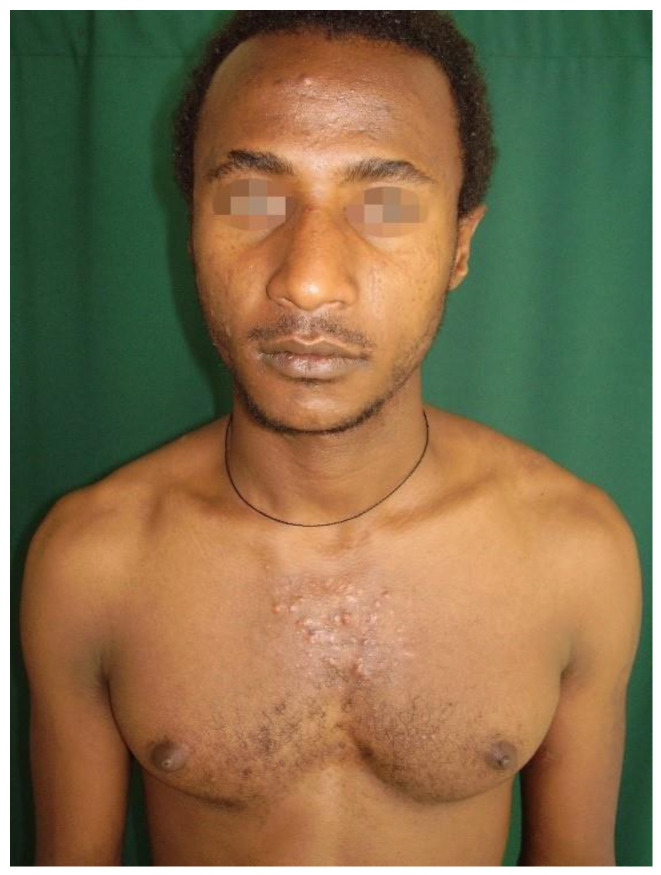
Acne vulgaris in a patient with highly pigmented skin. Reprinted with permission from [35].

**Figure 1:**
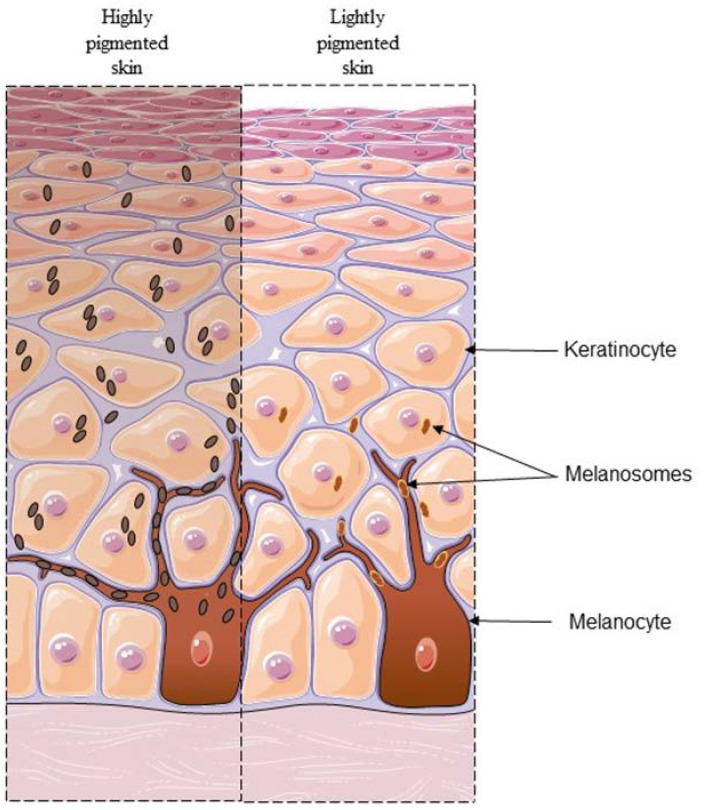
Differences described in the literature between two different tones of skin. In highly pigmented skin (**left**), melanosomes are big and abundant, and are transferred to keratinocytes as singly packaged organelles. In lightly pigmented skin (**right**), melanosomes are small and are transferred to keratinocytes as clusters in membrane-bound organelles.

**Figure 2:**
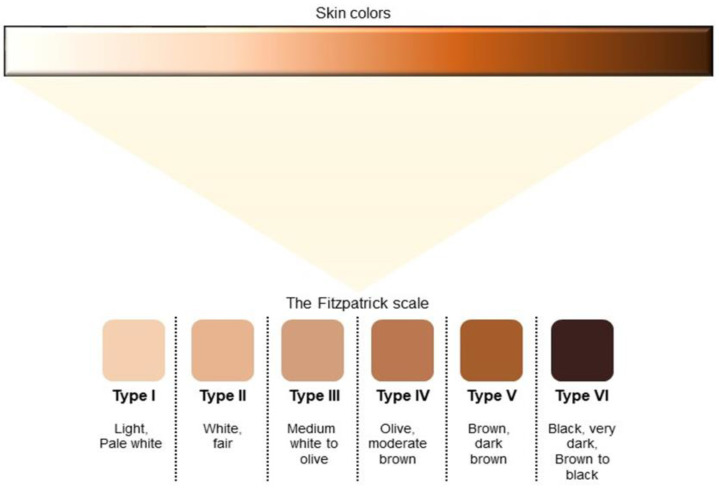
The Fitzpatrick scale of skin pigmentation tones. The scientific community is debating the propriety of summarizing so many shades of skin colour in only six classifications.

One of the most common pigmentary disorders is represented by Postinflammatory hyperpigmentation (PIH), which occurs after skin inflammation and has a higher incidence and greater severity in individuals with highly pigmented skin [33]. PIH is thought to be the default response to cutaneous injury that can occur after various dermatoses, exogenous stimuli, or dermatologic procedures. One of the major causes of PIH is represented by acne vulgaris, a multifactorial disease of the pilosebaceous unit with high prevalence in the world, regardless of the skin pigmentation of individuals. Effective therapy for people with acne vulgaris implies targeting the same pathological pathways in all patients, but individuals with highly pigmented skin may incur PIH even after acne lesions have resolved, as a consequence of acne inflammation or treatment, which may cause cutaneous irritation (Figure 3).

Nowadays, the knowledge about acne is increased in patients with highly pigmented skin, and also physicians are more educated about early effective treatments, more adequate skincare, and better sun prevention to avoid hyperpigmentation. Therefore, only better and appropriate management of patients can reduce the impact that acne has on the lives of people [34].

**Figure 4:**
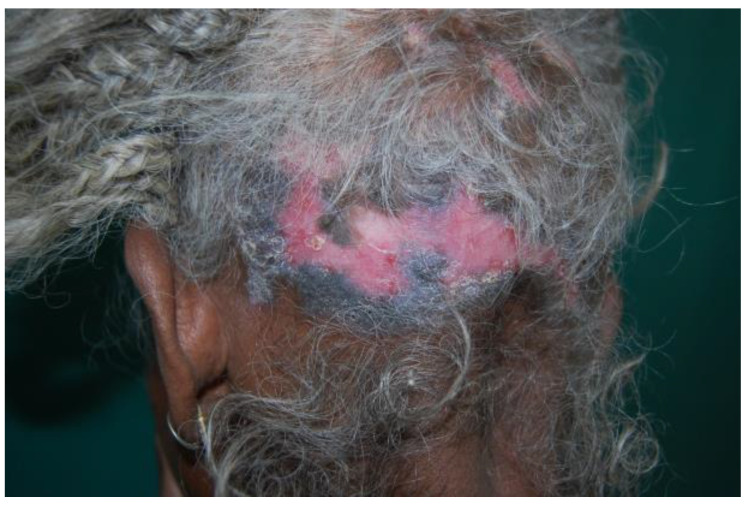
The hair and scalp of a patient with SLE. Reprinted with permission from [35].

PIH could also occur as a consequence of other disorders. Data in the literature show that one of the possible causes is represented by the autoimmune disease systemic lupus erythematosus (SLE). SLE is a complex and chronic autoimmune disease that affects many organs, including the skin. As for acne vulgaris, a higher incidence and major severity of SLE are observed in African American women and women in other minority population groups (Figure 4) [36]. There are also few studies about the incidence in the African population, but future data are necessary to highlight and support a geographical correlation for the manifestations of SLE.

### 6.3. Hyperpigmentation Disorders

**Figure 5:**
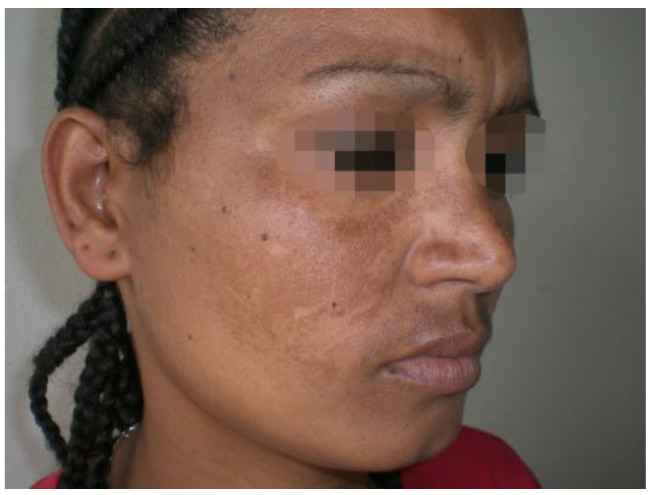
A woman with melasma. Reprinted with permission from [35].

The altered increase in skin pigmentation called hyperpigmentation could be the result of several skin alterations such as melasma. Melasma is an hyperpigmentary disorder, and it is more common in highly pigmented skin, in Asian and Hispanic populations living in places with high UVR exposure [37]. The pathology is a common disorder mainly affecting women of childbearing age, with asymptomatic light to dark brown spots and irregular edges in the photo-exposed areas, mainly on the face (Figure 5) [38]. Data in the literature report that melasma has an early onset in the life of fair-skinned people [39]. This may be a consequence of the photoprotective role of melanin which may delay the pathology onset in people with highly pigmented skin [11].

**Figure 6:**
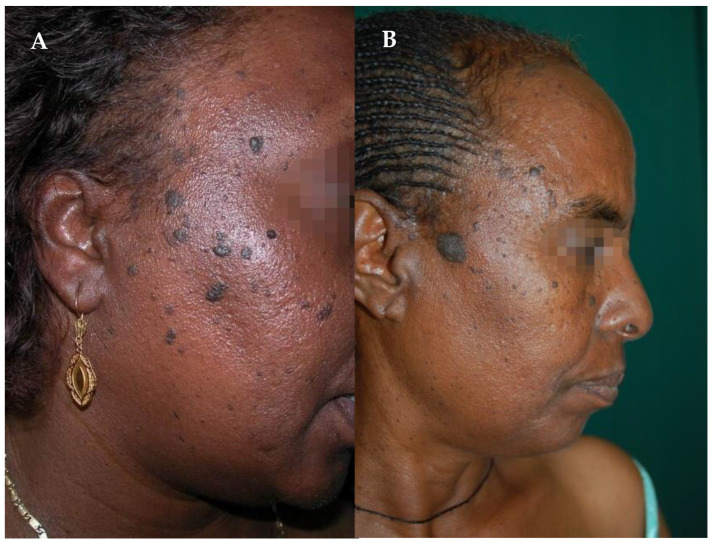
Two African women affected by (**A**) DPN and (**B**) SKs, respectively. Reprinted with permission from [35].

When it comes to hyperpigmentary disorders, it is also impossible not to mention Dermatosis papulosa nigra (DPN), a benign skin lesion type that may be seen mainly in people of African and Asian descent [40]. Because of the same pathological features, DPN is often categorized as a type of seborrheic keratosis (SKs) (Figure 6). Nonetheless, some dermatologists tend to distinct the two pathologies for several differences. DPN is common in African and Asian populations, with female predominance, whereas SKs are observed in all populations, with a predominance in fair-skinned individuals [41]. Furthermore, DPN is described with the appearance of multiple heavily pigmented papules that occur on sun-exposed areas, as over the face, neck, chest, and upper back, while SKs are diffused. On these bases, Xiao et al. suggest that UV exposure may have a role in the pathogenesis of [42]. However, the aetiology of DPN is still unknown, but data in the literature characterise this pathology.

### 6.4. Hypopigmentary Disorders

**Figure 7:**
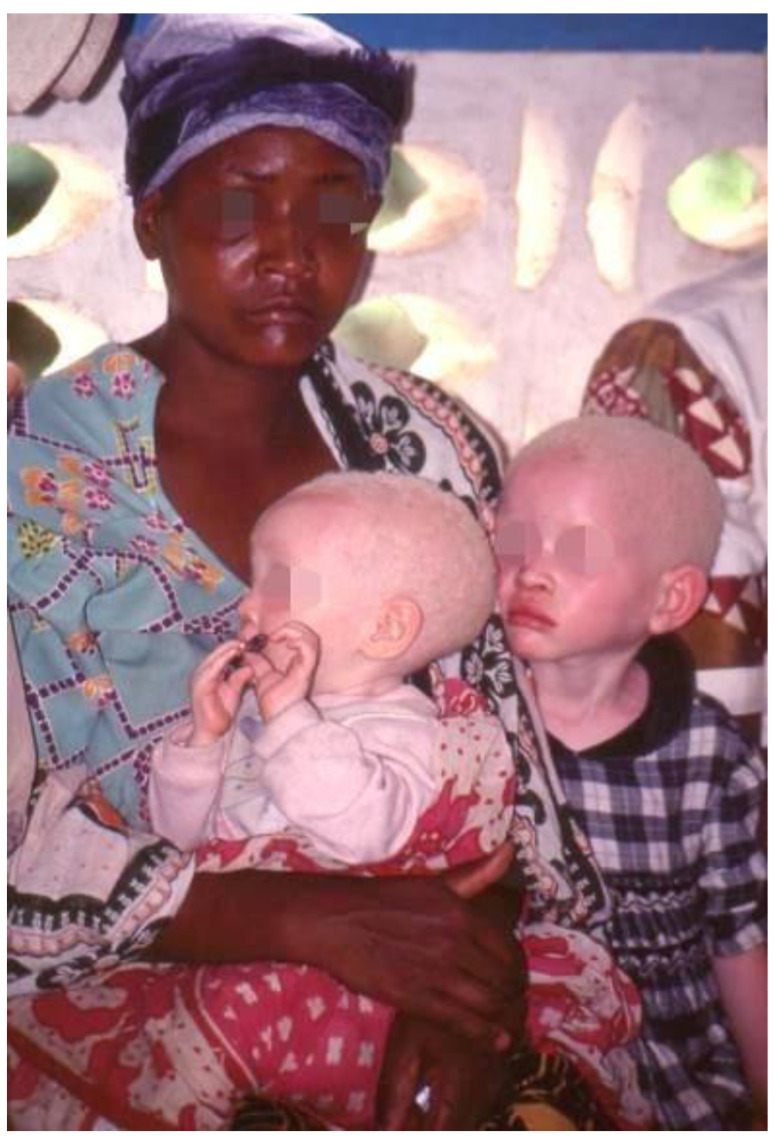
A mother with two children with albinism. Reprinted with permission from [35].

The first hypopigmentary condition that comes to mind is certainly albinism, a group of hereditary disorders produced by an impaired melanocyte system. Oculocutaneous albinism (OCA) represents the most common and visible type of albinism and is characterized by an autosomal recessive hereditability with a partial or complete loss of melanin in skin, hair and eyes (Figure 7). Moreover, OCA is associated with eye alterations such as photophobia, decreased visual acuity and nystagmus. The pathology has an important genetic component and has seven subtypes (OCA1-7) based on genetic mutation profiles which have different incidence among populations [43]. The OCA1 subtype occurs mainly in fair-skinned populations such as Europeans, and it is also the most common form of albinism for the Han Chinese population [44]; the OCA2 form has a major incidence in the African American population, whereas OCA3, also called “rufous albinism”, occurs in the African population, and is not reported in the fair-skinned populations [45]; OCA4 is the last most studied form of albinism and is reported to occur in approximately 18% of Japanese patients, but it results to be rare in other Asian populations [46]. OCA5/6/7 are rare forms of albinism and have few cases in the world. However, other studies on the disease are necessary to resolve the limits of the actual knowledge, especially because recent investigations highlighted new genetic aspects that have to be considered [47]. Therefore, beyond the severe physical symptoms, patients with albinism are victims of social barriers, with social stigmatisation and marginalization. In Africa, albinism is still misconceived and is correlated with myths and superstitions that lead to several social consequences, such as discrimination and violent attacks in some regions, with the sale of body parts on the black market [48]. Therefore, education for patients and good health care are needed, and it is also crucial to promote a cultural change in the concept of albinism in order to fight against discrimination and terrible violence directed at people with albinism. 

**Figure 8:**
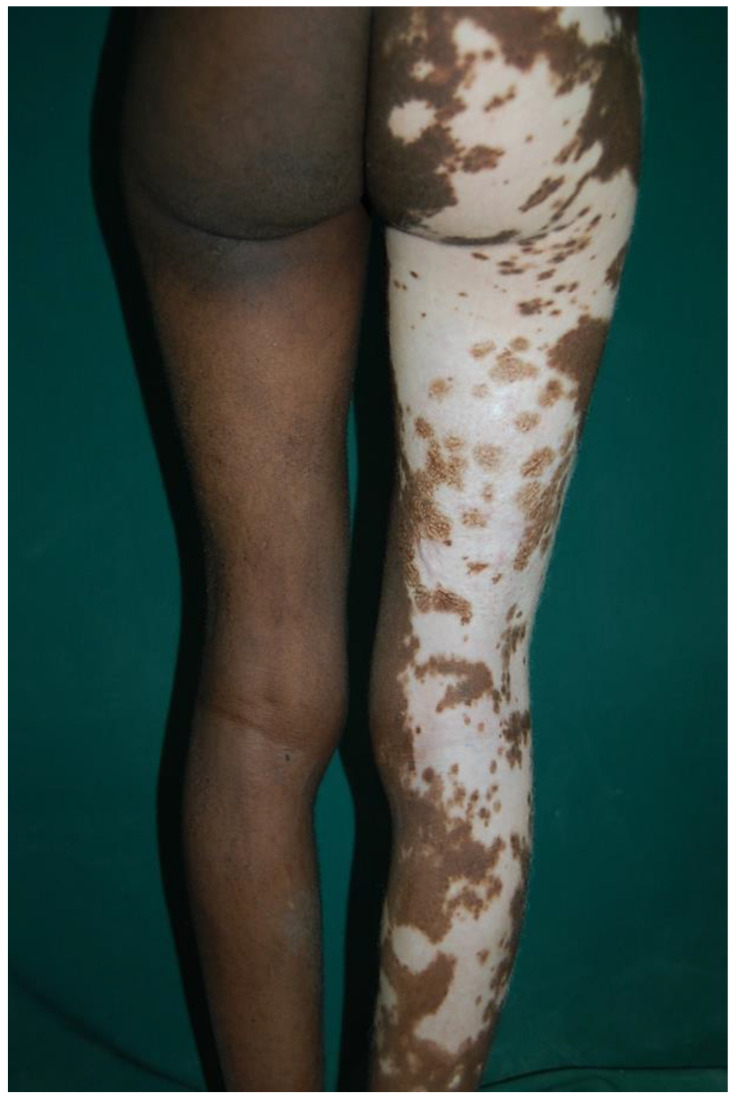
Legs of a woman affected by vitiligo. Reprinted with permission from [35].

Vitiligo is another important example of hypopigmentary disease. It is a multifactorial disease defined by a partial or total absence of skin pigmentation because of the activity decrease of melanocytes in the basal layer of the epidermis, mucosa or other organs. The typical manifestations of vitiligo are pale or milk-white macules or patches due to the selective destruction of melanocytes (Figure 8). The aetiology of vitiligo has yet to be fully understood, but it involves an interaction among several factors, such as the autoimmunity system and genetics [49]. The vitiligo incidence varies among geographic areas, but studies in this field still have limitations in sample size and localizations. In 2016, Zhang et al. evidenced a relatively high prevalence of the disease in African areas and in female patients [50]. Researchers also observed that this prevalence showed an inverse trend with age increment.

As is the case with albinism, culture and religion may influence the disease perception. A recent study highlighted that cultural and religious differences influence vitiligo awareness, so there is a strong necessity to improve the education of the community to avoid the marginalization of vitiligo patients [51].

### 6.5. Skin Cancers

**Figure 9:**
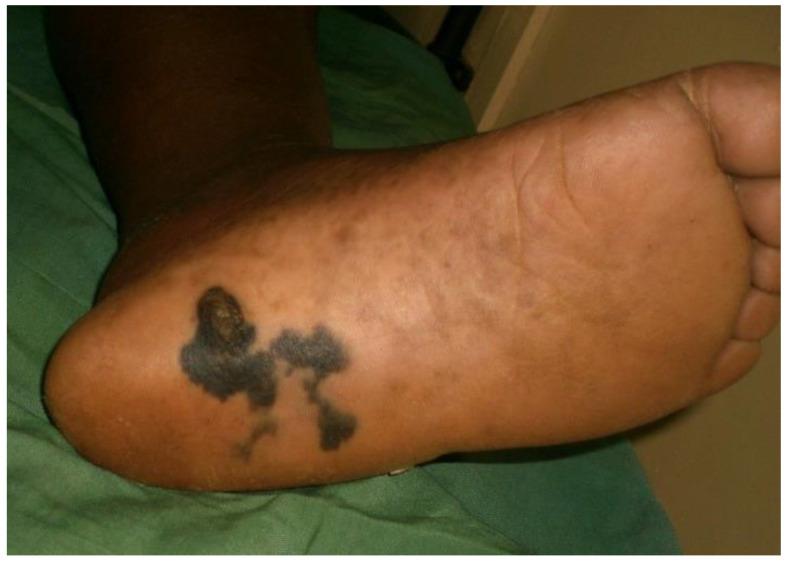
Plantar melanoma in an African patient. Reprinted with permission from [35].

UVR represent the predominant causal factor of skin photo-carcinogenesis. On the other hand, melanin protects against UVR, so the dermatological community has often wondered about a possible correlation between the severity of UVR-dependent DNA damage and skin pigmentation. Dermatologists mainly describe three skin cancers for an observed correlation with skin pigmentation: melanoma, basal cell carcinoma (BCC), and squamous cell carcinoma (SCC). Malignant melanoma has a higher incidence among individuals with lightly pigmented skin, while it is considered less frequent in African, Asian, and Hispanic populations [52]. Continuous UVR exposure appears to promote melanoma in people with lightly pigmented skin, whereas it does not appear to be a risk factor in people with highly pigmented skin [53]. Data in the literature mainly reports differences in the prognosis of melanoma among different populations. In people with highly pigmented skin, melanoma is often identified after spreading and the prognosis is consequently worse (Figure 9) [52]. Consequently, the 5-year melanoma survival rate is 66.7% for patients with highly pigmented skin compared with a 92.5% rate for patients with fair skin [54]. This divergence is an effect determined by numerous factors such as socio-economic differences, unequal access to health care, and health disparities. 

**Figure 10:**
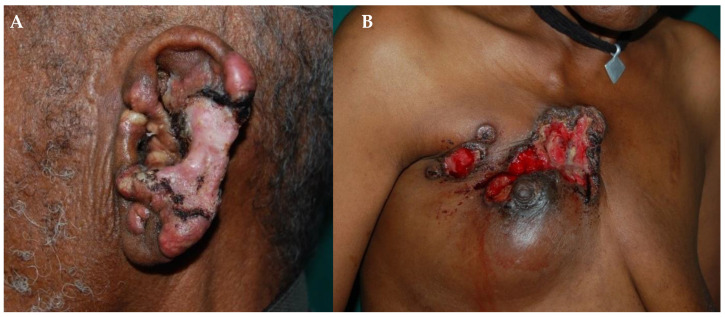
(**A**) BCC and (**B**) SCC in two Ethiopian patients. Reprinted with permission from [35].

BCC is a malignant cancer derived from basal cells of the skin. It is the most common skin cancer in Asian and Hispanic individuals and the second most common cancer in black individuals. BCC onset has been strictly related to sun-exposed areas. An estimated 90% of BCCs are found on the head–neck region in highly pigmented skin populations. Furthermore, people who live near the equator appear to have a higher risk of developing BCC (Figure 10A) [55]. Another common skin cancer is represented by SCC that is derived from squamous cells of epidermis and usually develops from a scar or burn. SCC represents the most common skin cancer in highly-pigmented-skin people (Figure 10B), and it is the second most common skin cancer in Hispanic and Asian individuals. Data in the literature show that these statistics highlight a strong relation between skin pigmentation, SCC onset, scarring processes, and inflammatory conditions such as burn scars, hidradenitis suppurativa, and ulcers [55].

Further studies are certainly needed to answer questions and contradictory results on the incidence statistics of BCC and SCC onset, because most of these investigations are based on limited sample sizes, so the results are not generalizable.

## 7. Dermatology and Inequalities

Even today, scientific language uses words that can be problematic and avoidable because of the shadow of racism bound to them. One example is the term “ethnicity” which can be considered ambiguous and a surrogate for race because it also includes non-biological factors such as culture and geographical areas. Furthermore, ethnicity does not include the inherent variability within the same population, incurring in an underestimation of specific scientific aspects. Thus, the ethnicity concept is a little inaccurate, and language should be improved with a more inclusive perspective. 

Unfortunately, the misuse of scientific language is exacerbated by real disparities in dermatology. Sometimes, the use of actual dermatological skin classifications such as the Fitzpatrick one perpetuates the false belief that individuals classified equally have all skin characteristics in common and share the same cutaneous cancer risks [14]. Moreover, clinicians as well as patients may misperceive the risk of skin cancer as lower in highly pigmented skin, thinking about stronger protection offered by the amount of melanin. This underestimation could partially explain the late skin cancer diagnoses in individuals with highly pigmented skin, with consequent worse outcomes [56]. Another important factor to consider is the tendency of some dermatologists to visit patients with an “Eurocentric approach”, not seeing or misinterpreting important pathological signals in patients with highly pigmented skin [14]. Moreover, studies in the literature highlight that some dermatologists, mainly those with a poor experience, are not sufficiently prepared to identify and treat the disorders of skin tones different than fair ones, where clinical manifestation can be different. Investigations in the literature show that this insufficient training is also due to the lack of illustrations in the textbooks of pathologies in patients of all types of skin [57]. One updated analysis has shown that only one dermatological text out of the six commonly used had an increase of more than 1% in pictures with highly pigmented skin in the past 15 years [58]. Thus, the same pathology may be represented variously in patients with different skin pigmentation and the dermatologist might be unable to recognize the dermatological disorder without a supplement to their curriculum. On these bases, common dermatological conditions can be misdiagnosed or undiagnosed, resulting in prolonged patient suffering and worse outcomes. 

To address the lack of reference photos of cutaneous diseases on highly pigmented skin, in 2018, Ellen Buchanan Weiss, the mother of a neonatal patient, created the Instagram project “Brown Skin Matters” (@brownskinmatters), a community-sourced database of photos of several dermatological conditions on non-fair skin. A web database or account cannot replace medical education, but it could be a helpful resource for dermatologists.

The problem is exacerbated by the fact that in medicine, and especially in dermatology, only a small percentage of physicians have high-pigmented skin. Recent statistics have reported, for example, that in the United States, only 3% of dermatologists have highly pigmented skin. This phenomenon, together with intrinsic racism, is also associated with the education level of many patients on disease prevention, healthcare, and exposure to environmental pollutants and irritants [59].

In recent years, the scientific community is trying to overcome all of these issues bound to health disparities. The British Association of Dermatologists is an example of this: it is a UK professional membership body for dermatologists dedicated to education, practice, research, and equality in healthcare. Many excellent researchers are contributing to this goal, as evidenced, for example, by the aforementioned study by Dadzie et al. [28], which shows a growing awareness of the need to achieve equality in the field of dermatology.

## 8. Conclusions

Pigmentation determines the unique characteristics of the skin, but on the other hand, the differences often lead to prejudices and preconceptions. Dermatology must guarantee the same attention and care to all skin types. The training of young physicians has to be improved with specific courses and workshops and with an educational approach with more pictures of skin manifestations in non-fair skin in medical textbooks. Today, dermatological education could also be ameliorated with the help of online resources, which are characterized by easier access to the different clinical manifestations among the various skin tones.

The perception of skin colour as a social determinant still impacts healthcare today. We believe that hard work is needed to eliminate the roots of this distorted view by focusing on improving access to services and promoting health and cultural education. Although the process may be slow, the scientific community is working to overcome the barriers of scientific racism and health inequalities, but there is still a long way to go.

## Data Availability

Not applicable.

## References

[1] Trawalter S., Bart-Plange D., Hoffman K.M. (2020). A Socioecological Psychology of Racism: Making Structures and History More Visible. Curr. Opin. Psychol..

[2] Diogo R. (2018). Links between the Discovery of Primates and Anatomical Comparisons with Humans, the Chain of Being, Our Place in Nature, and Racism. J. Morphol..

[3] Bergman J. (2020). Louis Agassiz and Alexander Winchell: Two Case Histories of Creationists Who Illustrate That Rejecting Genesis Influences the Acceptance of Racism. ARJ.

[4] Guglielmo J., Salvatore S. (2003). Are Italians White? How Race Is Made in America.

[5] Caspari R. (2018). Race, Then and Now: 1918 Revisited. Am. J. Phys. Anthropol..

[6] Wakamatsu K., Kavanagh R., Kadekaro A.L., Terzieva S., Sturm R.A., Leachman S., Abdel-Malek Z., Ito S. (2006). Diversity of Pigmentation in Cultured Human Melanocytes Is Due to Differences in the Type as Well as Quantity of Melanin. Pigment Cell Res..

[7] D’Alba L., Shawkey M.D. (2019). Melanosomes: Biogenesis, Properties, and Evolution of an Ancient Organelle. Physiol. Rev..

[8] Hurbain I., Romao M., Sextius P., Bourreau E., Marchal C., Bernerd F., Duval C., Raposo G. (2018). Melanosome Distribution in Keratinocytes in Different Skin Types: Melanosome Clusters Are Not Degradative Organelles. J. Investig. Dermatol..

[9] Thong H., Jee S., Sun C., Boissy R.E. (2003). The Patterns of Melanosome Distribution in Keratinocytes of Human Skin as One Determining Factor of Skin Colour. Br. J. Dermatol..

[10] Micillo R., Panzella L., Koike K., Monfrecola G., Napolitano A., d’Ischia M. (2016). “Fifty Shades” of Black and Red or How Carboxyl Groups Fine Tune Eumelanin and Pheomelanin Properties. Int. J. Mol. Sci..

[11] Del Bino S., Duval C., Bernerd F. (2018). Clinical and Biological Characterization of Skin Pigmentation Diversity and Its Consequences on UV Impact. Int. J. Mol. Sci..

[12] Swope V., Alexander C., Starner R., Schwemberger S., Babcock G., Abdel-Malek Z.A. (2014). Significance of the Melanocortin 1 Receptor in the DNA Damage Response of Human Melanocytes to Ultraviolet Radiation. Pigment Cell Melanoma Res..

[13] Shulpekova Y., Nechaev V., Kardasheva S., Sedova A., Kurbatova A., Bueverova E., Kopylov A., Malsagova K., Dlamini J.C., Ivashkin V. (2021). The Concept of Folic Acid in Health and Disease. Molecules.

[14] Norton H.L. (2021). The Color of Normal: How a Eurocentric Focus Erases Pigmentation Complexity. Am. J. Hum. Biol..

[15] Jablonski N.G. (2021). The Evolution of Human Skin Pigmentation Involved the Interactions of Genetic, Environmental, and Cultural Variables. Pigment Cell Melanoma Res..

[16] David-Barrett T., Dunbar R.I. (2016). Bipedality and Hair Loss in Human Evolution Revisited: The Impact of Altitude and Activity Scheduling. J. Hum. Evol..

[17] Kimbel W.H., Villmoare B. (2016). From Australopithecus to Homo: The Transition That Wasn’t. Philos. Trans. R. Soc. Lond. B. Biol. Sci..

[18] Wheeler P.E. (1992). The Thermoregulatory Advantages of Large Body Size for Hominids Foraging in Savannah Environments. J. Hum. Evol..

[19] Christophers E., Schroder J. (2022). Evolution of Innate Defense in Human Skin. Exp. Dermatol..

[20] Carotenuto F., Tsikaridze N., Rook L., Lordkipanidze D., Longo L., Condemi S., Raia P. (2016). Venturing Out Safely: The Biogeography of Homo Erectus Dispersal Out of Africa. J. Hum. Evol..

[21] Hershkovitz I., Weber G.W., Quam R., Duval M., Grun R., Kinsley L., Ayalon A., Bar-Matthews M., Valladas H., Mercier N. (2018). The Earliest Modern Humans Outside Africa. Science.

[22] Feng Y., McQuillan M.A., Tishkoff S.A. (2021). Evolutionary Genetics of Skin Pigmentation in African Populations. Hum. Mol. Genet..

[23] Holubar K. (1996). What is a Caucasian?. J. Investig. Dermatol..

[24] Bhopal R. (2007). The Beautiful Skull and Blumenbach’s Errors: The Birth of the Scientific Concept of Race. BMJ.

[25] Fitzpatrick T.B. (1988). The Validity and Practicality of Sun-Reactive Skin Types I through VI. Arch. Dermatol..

[26] Ware O.R., Dawson J.E., Shinohara M.M., Taylor S.C. (2020). Racial Limitations of Fitzpatrick Skin Type. Cutis.

[27] Sharma V.K., Gupta V., Jangid B.L., Pathak M. (2018). Modification of the Fitzpatrick system of skin phototype classification for the Indian population, and its correlation with narrowband diffuse reflectance spectrophotometry. Clin. Exp. Dermatol..

[28] Dadzie O.E., Sturm R.A., Fajuyigbe D., Petit A., Jablonski N.G. (2022). The Eumelanin Human Skin Colour Scale: A Proof-of-Concept Study. Br. J. Dermatol..

[29] Nakashima Y., Wada K., Yamakawa M., Nagata C. (2022). Validity of self-reported skin color by using skin color evaluation scale. Skin Res. Technol..

[30] Dass A. Humanae Project. https://angelicadass.com/photography/humanae/.

[31] Ames B.N., Grant W.B., Willett W.C. (2021). Does the High Prevalence of Vitamin D Deficiency in African Americans Contribute to Health Disparities?. Nutrients.

[32] Jablonski N.G., Chaplin G. (2012). Human Skin Pigmentation, Migration and Disease Susceptibility. Philos. Trans. R. Soc. Lond. B Biol. Sci..

[33] Lawrence E., Al Aboud K.M. (2022). Postinflammatory Hyperpigmentation. Statpearls.

[34] Chiang C., Ward M., Gooderham M. (2022). Dermatology: How to Manage Acne in Skin of Colour. Drugs Context.

[35] Morrone A. (2022). Atlante di Dermatologia Tropicale.

[36] Gomez-Puerta J.A., Barbhaiya M., Guan H., Feldman C.H., Alarcon G.S., Costenbader K.H. (2015). Racial/Ethnic Variation in All-Cause Mortality among United States Medicaid Recipients with Systemic Lupus Erythematosus: A Hispanic and Asian Paradox. Arthritis Rheumatol..

[37] Taylor S.C. (2003). Epidemiology of Skin Diseases in Ethnic Populations. Dermatol. Clin..

[38] Cario M. (2019). How Hormones May Modulate Human Skin Pigmentation in Melasma: An In Vitro Perspective. Exp. Dermatol..

[39] Ortonne J.P., Arellano I., Berneburg M., Cestari T., Chan H., Grimes P., Hexsel D., Im S., Lim J., Lui H. (2009). A Global Survey of the Role of Ultraviolet Radiation and Hormonal Influences in the Development of Melasma. J. Eur. Acad. Dermatol. Venereol..

[40] Metin S.A., Lee B.W., Lambert W.C., Parish L.C. (2017). Dermatosis Papulosa Nigra: A Clinically and Histopathologically Distinct Entity. Clin. Dermatol..

[41] Alapatt G.F., Sukumar D., Bhat M.R. (2016). A Clinicopathological and Dermoscopic Correlation of Seborrheic Keratosis. Indian J. Dermatol..

[42] Xiao A., Muse M.E., Ettefagh L. (2022). Dermatosis Papulosa Nigra. Statpearls.

[43] Arveiler B., Lasseaux E., Morice-Picard F. (2017). Clinical and Genetic Aspects of Albinism. Presse Med..

[44] Wei A., Yang X., Lian S., Li W. (2011). Implementation of an Optimized Strategy for Genetic Testing of the Chinese Patients with Oculocutaneous Albinism. J. Dermatol. Sci..

[45] Rooryck C., Morice-Picard F., Elcioglu N.H., Lacombe D., Taieb N., Arveiler B. (2008). Molecular Diagnosis of Oculocutaneous Albinism: New Mutations in the OCA1-4 Genes and Practical Aspects. Pigment Cell Melanoma Res..

[46] Inagaki K., Suzuki T., Shimizu H., Ishii N., Umezawa Y., Tada J., Kikuchi N., Takata M., Takamori K., Kishibe M. (2004). Oculocutaneous Albinism Type 4 Is One of the Most Common Types of Albinism in Japan. Am. J. Hum. Genet..

[47] Yang Q., Yi S., Li M., Xie B., Luo J., Wang J., Rong X., Zhang Q., Qin Z., Hang L. (2019). Genetic Analyses of Oculocutaneous Albinism Types 1 and 2 with Four Novel Mutations. BMC Med. Genet..

[48] Kromberg J.G.R., Kerr R. (2022). Oculocutaneous Albinism in Southern Africa: Historical Background, Genetic, Clinical and Psychosocial Issues. Afr. J. Disabil..

[49] Ezzedine K., Eleftheriadou V., Whitton M., van Geel N. (2015). Vitiligo. Lancet.

[50] Zhang Y., Cai Y., Shi M., Jiang S., Cui S., Wu Y., Gao X., Chen H. (2016). The Prevalence of Vitiligo: A Meta-Analysis. PLoS ONE.

[51] Juntongjin P., Abouelsaad S., Sugkraroek S., Taechakraichana N., Lungchukiet P., Nuallaong W. (2022). Awareness of Vitiligo among Multi-Ethnic Populations. J. Cosmet. Dermatol..

[52] McKenzie S., Brown-Korsah J.B., Syder N.C., Omar D., Taylor S.C., Elbuluk N. (2022). Variations in Genetics, Biology, and Phenotype of Cutaneous Disorders in Skin of Color. Part II: Differences in Clinical Presentation and Disparities in Cutaneous Disorders in Skin of Color. J. Am. Acad. Dermatol..

[53] Lopes F.C.P.S., Sleiman M.G., Sebastian K., Bogucka R., Jacobs E.A., Adamson A.S. (2021). UV Exposure and the Risk of Cutaneous Melanoma in Skin of Color: A Systematic Review. JAMA Dermatol..

[54] Howlader N., Noone A., Krapcho M., Miller D., Brest A., Yu M., Ruhl J., Tatalovich Z., Mariotto A., Lewis D. (2015). SEER Cancer Statistics Review, 1975–2016.

[55] Hogue L., Harvey V.M. (2019). Basal Cell Carcinoma, Squamous Cell Carcinoma, and Cutaneous Melanoma in Skin of Color Patients. Dermatol. Clin..

[56] Buster K.J., Stevens E.I., Elmets C.A. (2012). Dermatologic Health Disparities. Dermatol. Clin..

[57] Perlman K.L., Williams N.M., Egbeto I.A., Gao D.X., Siddiquee N., Park J.H. (2021). Skin of Color Lacks Representation in Medical Student Resources: A Cross-Sectional Study. Int. J. Womens Dermatol..

[58] Adelekun A., Onyekaba G., Lipoff J.B. (2021). Skin Color in Dermatology Textbooks: An Updated Evaluation and Analysis. J. Am. Acad. Dermatol..

[59] Perlman K.L., Klein E.J., Park J.H. (2020). Racial Disparities in Dermatology Training: The Impact on Black Patients. Cutis.

